# Psychiatric symptoms, burnout and associated factors in psychiatry residents

**DOI:** 10.47626/2237-6089-2020-0040

**Published:** 2021-10-22

**Authors:** Gabriela Massaro Carneiro Monteiro, Grasiela Marcon, Glen Owens Gabbard, Fernanda Lucia Capitanio Baeza, Simone Hauck

**Affiliations:** 1 Programa de Pós-Graduação em Psiquiatria e Ciências do Comportamento Universidade Federal do Rio Grande do Sul Porto Alegre RS Brazil Programa de Pós-Graduação em Psiquiatria e Ciências do Comportamento, Universidade Federal do Rio Grande do Sul (UFRGS), Porto Alegre, RS, Brazil.; 2 Departamento de Psiquiatria Universidade Federal da Fronteira Sul Chapecó SC Brazil Departamento de Psiquiatria, Universidade Federal da Fronteira Sul (UFFS), Chapecó, SC, Brazil.; 3 Baylor College of Medicine Houston TX USA Baylor College of Medicine, Houston, TX, USA.

**Keywords:** Burnout, medical education, mental health, psychiatry residents and work environment

## Abstract

**Introduction:**

Mental health in training physicians is a growing issue. The aim of this study was to investigate emotional distress in psychiatry residents.

**Method:**

This web-based survey evaluated 115 (62%) psychiatry residents in training in the Brazilian State of Rio Grande do Sul. The DSM-5 Self-Rated Level 1 Cross-Cutting Symptom Measure-Adult, the Patient Health Questionnaire-2, the Alcohol Use Disorders Identification Test-concise, and the Maslach Burnout Inventory were all administered. Linear regression models were estimated with burnout dimensions as dependent variables.

**Result:**

Positive screening rates were 53% for anxiety, 35.7% for somatization, 16.5% for depression, and 7% for suicidal ideation. Half of the male residents were at risk of alcohol abuse and dependence. Regarding burnout, 60% met criteria for emotional exhaustion, 54.8% for depersonalization, and 33% for low personal accomplishment. The most consistent risk factors were the nature of the relationships with preceptors, relations to the institutions themselves, age, and the quality of relationships with family.

**Conclusion:**

Besides disconcerting rates of psychiatric symptoms, the study revealed that characteristics of the workplace (i.e., the nature of relationships with preceptors and relations to the institution) can be regarded as potential targets for development of interventions aimed at improving mental health during training periods.

## Introduction

Mental health in physicians is an emergent issue nowadays and the occurrence of burnout is a growing concern; often referred to as an epidemic phenomenon in the literature.^1,2^ In 2019, 44-47% of US physicians described themselves as feeling burned out or at least reported symptoms of burnout.^3,4^ Mihailescu & Neiterman^5^ published a review in 2019 analyzing the extant literature on mental health concerns in physicians and physicians-in-training in North America. They found that, on average, the literature suggests that burnout and mental health concerns affect 30-60% of all physicians and residents. There was some overlap among papers discussing burnout, depression, and suicidal ideation, suggesting that work-related stress may lead to the emergence of more serious mental health problems as well as addiction and substance abuse. Residency training was shown to produce the highest rates of burnout. In addition, they found that papers discussing causes of deterioration of mental health in physicians (20%) and prevalence of mental illness (16%) were the least common.

Burnout is a syndrome that includes emotional exhaustion, depersonalization, and low sense of personal accomplishment. Emotional exhaustion (EE) is described as lack of enthusiasm and energy, leading to a feeling of resource depletion. Depersonalization (DP) is defined as emotional insensitivity, characterized by disillusionment with the service provided, culminating in dehumanization and impersonal treatment of patients and colleagues. Low sense of personal accomplishment (PA) at work refers to a sense of inadequacy and low self-esteem connected to a belief that professional goals have not been met.^6,7^ In 2018, the World Health Organization (WHO) recognized burnout as an occupational phenomenon and included it in the 11th Revision of the International Classification of Diseases (ICD-11).^8^

Medical training can be associated with uncertainties about the future, feelings of insecurity, high levels of responsibility, and high workload. Current studies show that the prevalence of burnout in residents is about 25-75%, varying by specialty, country, and methods of measurement.^9,10^ Research investigating psychiatry residents suggests that there is a 23-36% prevalence of burnout^11-13^ and reveals associations with various demographic, learner, and workplace factors. These include non-parental status, being married, increased workload, insufficient rest, lack of supervision at work, being in early years of training, lower priority of psychiatry as career choice, decreased empathic capacity, poor coping skills, increased medical errors, more stressors, and low self-efficacy.^10,12-18^ Some studies with psychiatry residents have revealed psychic distress among participants, namely symptoms of depression, anxiety, suicidal ideation, and use of psychotropic medications.^12,19^ Nevertheless, more specific factors of the work environment such as the nature of relationships within the institutions are still poorly understood.

Despite the increased interest in burnout and mental health in general, studies in psychiatry residents are scarce. Furthermore, there is a lack of studies investigating specific factors of the work environment that may be related to emotional distress in this specific population. The aim of this study was to investigate emotional distress in psychiatry residents, especially burnout, psychiatric symptoms, and the role of the work environment.

## Methods

In this cross-sectional study, all psychiatry residents in training in the Brazilian State of Rio Grande do Sul (n = 185) were invited to participate over a period of one month at the end of 2018. Data were collected with an online questionnaire sent by e-mail. We chose an electronic questionnaire because of its ease of response, and because it has the potential advantage of enhancing reliability by augmenting the perception of anonymity. Subjects could only access the questionnaire if they had agreed to the online Informed Consent Form. After completion, the questionnaire provided telephone and electronic contact information for suicide prevention and support centers located in Brazil. The study was approved by the Hospital de Clínicas de Porto Alegre ethics committee (Porto Alegre, Brazil) (protocol CAAE 70231617.6.0000.5327).

### Survey instruments

The online questionnaire included questions regarding sociodemographic data, personal information, career status, workload, and mental health variables, including current psychiatric treatment, harassment, discrimination, and abuse at the workplace, alcohol and drug use, sleep patterns, and quality of relationships with family and friends.

Burnout level was measured by means of the Portuguese version of the Maslach Burnout Inventory - Human Services Survey (MBI-HSS).^7^ The MBI-HSS measures burnout on three subscales: emotional exhaustion (EE), depersonalization (DP), and sense of personal accomplishment (PA). The scale comprises 22 items, 9 related to EE, 5 to DP, and 8 to PA. These three dimensions are related to each other but independent. There is an important debate in the literature regarding MBI cut-off points. Therefore, we defined the prevalence of each dimension using the most common cutoffs presented in the literature (EE ≥ 27; DP ≥ 10; PA ≤ 33), but we decided to use continuous scores for the three burnout dimensions when analyzing them as dependent factors to evaluate risk factors.^20^

The Work Environment Evaluation Instrument (WEEI) was used to assess the relationships with superiors and peers and relations to the institutions themselves. The WEEI was developed and validated by Monteiro et al.^21^ It is scored on a five-point Likert scale, where 0 corresponds to “Totally false” and 4 corresponds to “Totally true.” Five items evaluate the relationship with preceptors/supervisors, three with colleagues/peers, and three assess relations to the institutions themselves. The items evaluate aspects like feeling comfortable asking for help, feeling heard and helped versus feeling pressured by preceptors/supervisors, the feeling of belonging, and the presence of a collaborative atmosphere in the institution, and also the perception of support from peers. Cutoffs suggested by the authors in the validation study define the environment as healthy (> 32 points), risky (23-31 points), or toxic (< 22 points). The WEEI was originally developed and validated in Brazilian Portuguese.^21^ The WEEI’s total and dimension scores and the environment category (i.e., healthy, risky, or toxic) were all related to burnout symptoms.^22^

The DSM-5 Self-Rated Level 1 Cross-Cutting Symptom Measure-Adult was used to assess presence of psychiatric symptoms. This is a general screening measure for the main DSM 5 diagnostic categories that has been validated in Brazilian Portuguese.^23^

The Patient Health Questionnaire-2 (PHQ-2) was administered to assess depressive symptoms. The PHQ-2 consists of two questions related to symptoms of depression during the past two weeks. Scores on the PHQ-2 range from 0 to 6, where 0 indicates no cardinal depressive symptoms and 6 indicates feeling depressed and anhedonic essentially every day. A score of 3 or higher on the PHQ-2 is considered a positive screening result for depression. The PHQ-2 has sensitivity of 83% and specificity of 92% for diagnosis of a depressive episode.^24^

The AUDIT-C was used to evaluate alcohol use.^25^ This is a 3-item screening instrument that can help to identify persons who are high-risk drinkers or have active alcohol use disorder (including alcohol abuse or dependence). It is a modified version of the 10 questions AUDIT instrument.^26^ The AUDIT-C has sensitivity of 79% and specificity of 56% in men (score ≥ 4) and sensitivity of 80% and specificity of 87% in women (score ≥ 3) for identifying patients with active alcohol abuse or dependence.^24^ For men, scores of 0 to 3 were considered low risk; from 4 to 5 points, moderate risk; from 6 to 7 points, high risk, and from 8 to 12 points, severe risk. For women, scores of 0 to 2 were considered low risk; from 3 to 5 points, moderate risk; from 6 to 7 points, high risk, and from 8 to 12 points, severe risk. The AUDIT-C has been validated in Brazilian Portuguese.^27^

### Outcome

Levels of EE, DP, and PA were used as dependent variables in an analysis to identify risk factors for each burnout dimension.

### Statistical analysis

The Statistical Package for Social Sciences (SPSS) version 18 was used to analyze the data. The normality of data was evaluated using the Kolmogorov-Smirnov test and graphical analysis. Descriptive analyses were reported as means and standard deviations (SD), medians, and interquartile ranges (IQR), or absolute and relative frequencies. According to the distribution of burnout dimension scores, the difference between groups was evaluated by means of the Mann-Whitney U test and Kruskal-Wallis one-way analysis of variance (ANOVA). The post-hoc test used was the Dunn test with Bonferroni correction. Spearman correlation coefficients were calculated to estimate associations between variables. A linear regression model was estimated for each of the three burnout dimensions (dependent variables), including all potential risk factors that were associated with each outcome (p < 0.05) in the univariate analysis. We performed risk estimate tests to evaluate the environment category and burnout dimensions. A significance level of 5% (p ≤ 0.05) was adopted for all statistical tests. All tests were two-tailed.

## Results

One hundred and eight-five psychiatry residents were invited to answer the questionnaire, 132 of them answered it, and 115 (62%) were included in our sample, after excluding 17 individuals because they did not answer all the questions. Table 1 lists sociodemographic characteristics of the participants as well as information about physical exercise according to WHO standards (150 minutes of moderate-intensity physical activity throughout the week, or at least 75 minutes of vigorous-intensity physical activity throughout the week), relationship with family and friends, and satisfaction with one’s sexual life. Table 1 also describes features of participants’ work and some of their clinical psychiatric characteristics. Sixty-nine individuals (60%) met criteria for EE, 63 (54.8%) for DP, and 38 (33%) for low sense of PA, according to the cut-off points used (EE ≥ 27; DP ≥ 10; PA ≤ 33).

**Table 1:** Sociodemographic and clinical characteristics of participants

	Total	Male	Female

	(n = 115)	(n = 56)	(n = 59)
Age, mean (SD)	29.34 (3.5)	29.02 (3.3)	29.64 (3.7)
			
Sex	-	56 (48.7)	59 (51.3)
Heterosexual	88 (76.5)	38 (33)	50 (43.5)
Has partner	89 (77.4)	43 (37.4)	46 (40)
Has children	9 (7.8)	5 (4.4)	4 (3.4)
Living alone	50 (43.5)	26 (22.6)	24 (20.9)
Monthly family income			
> US$ 3875	39 (33.9)	19 (16.5)	20 (17.4)
US$ 1917-3875	32 (27.8)	15 (13)	17 (14.8)
US$ 775-1937	33 (28.7)	17 (14.8)	16 (13.9)
US$ 387-775	11 (9.6)	5 (4.3)	6 (5.3)
Financial help	73 (63.5)	35 (30.5)	38 (33)
Exercise according to WHO	45 (39.1)	25 (21.7)	20 (17.4)
Sex life is satisfactory	67 (58.3)	36 (31.3)	31 (27)
Sleep hours, mean (SD)	6.63 (0.986)	6.63 (0.94)	6.63 (1.03)
Sleep quality			
Bad	10 (8.7)	5 (4.35)	5 (4.35)
Regular	48 (41.7)	28 (24.3)	20 (17.4)
Good	39 (33.9)	15 (13)	24 (20.9)
Great	16 (13.9)	7 (6.1)	9 (7.8)
Excellent	2 (1.7)	1 (0.86)	1 (0.86)
Relationship with family			
Bad	1 (0.86)	0	1 (0.86)
Regular	21 (18.3)	16 (13.95)	5 (4.35)
Good	36 (31.3)	19 (16.5)	17 (14.8)
Great	39 (33.9)	13 (11.3)	26 (22.6)
Excellent	18 (15.7)	8 (7)	10 (8.7)
Relationship with friends			
Bad	2 (1.7)	0	2 (1.7)
Regular	14 (12.2)	6 (5.2)	8 (7)
Good	42 (36.5)	24 (20.9)	18 (15.6)
Great	37 (32.2)	19 (16.6)	18 (15.6)
Excellent	20 (17.4)	7 (6.1)	13 (11.3)
Residence year			
1	36 (31.3)	16 (13.9)	20 (17.4)
2	36 (31.3)	16 (13.9)	20 (17.4)
3	43 (37.4)	24 (20.9)	19 (16.5)
Other employment	63 (54.8)	32 (27.8)	31 (27)
Works at night	31 (27)	18 (15.6)	13 (11.4)
Works weekends	38 (33)	19 (16.5)	19 (16.5)
Thought of giving up	34 (29.6)	15 (13,1)	19 (16.5)
Satisfied with the profession			
Very satisfied	21 (18.3)	9 (7.8)	12 (10.5)
Satisfied	77 (67)	41 (35.6)	36 (31.4)
Unsatisfied	13 (11.3)	5 (4.3)	8 (7)
Very unsatisfied	4 (3.5)	1 (0.87)	3 (2.63)
Support from a preceptor	99 (86.1)	49 (42.6)	50 (43.5)
Preceptor abuse/harassment	66 (57.4)	30 (26.1)	36 (31.3)
Academic impact	49 (42.6)*	23 (20)	26 (22.6)
Years working as physician, mean (SD)	3.67 (3.2)	3.93 (3.7)	3.42 (2.8)
Hours per week of practice in residency, mean (SD)	46.45 (13.0)	47.41 (13.4)	45.54 (12.8)
Hours per week of study in residency, mean (SD)	9 (5.7)	9 (5.1)	8.92 (6.2)
Hours per week of leisure, mean (SD)	15.37 (14.3)	16.86 (15.9)	13.97 (12.5)
Hours per week of work unrelated to residency, mean (SD)	8 (10.35)	8.48 (10.1)	7.61 (10.6)
Psychotropic use			
Yes	66 (57.4)	31 (27)	35 (30.4)
With medical prescription	44 (38.3)*	19 (16.5)	25 (21.8)
Self-prescription, or friends	22 (19.1)*	12 (10.4)	10 (8.7)
Psychiatric or psychotherapeutic treatment			
Yes, psychiatric and psychotherapeutic treatment	33 (28.7)	12 (10.5)	21 (18.2)
Yes, psychotherapeutic treatment	40 (34.8)	20 (17.4)	20 (17.4)
Yes, just psychiatric treatment	7 (6.1)	3 (2,7)	4 (3.4)
No	35 (42.4)	21 (18.2)	14 (24.2)
Screened positive for			
Anxiety	61 (53)	31 (27)	30 (26)
Somatization	41 (35.7)	16 (13.9)	25 (21.8)
PHQ 2	19 (16.5)	7 (6.1)	12 (10.4)
Personality functioning	36 (31.3)	16 (13.9)	20 (17.4)
Anger	31 (27)	12 (10.4)	19 (16.6)
Mania	14 (12.2)	7 (6.1)	7 (6.1)
Repetitive thoughts and behaviors	9 (7.8)	5 (4.4)	4 (3.4)
Dissociation	4 (3.5)	2 (1.75)	2 (1.75)
Suicide ideation	8 (7)	4 (3.5)	4 (3.5)
Tobacco use	20 (17.4)	15 (13)	5 (4.4)
Marijuana use 3 months	13 (11.3)	6 (5.2)	7 (6.1)
AUDIT-C			
Low risk	-	28 (50)	49 (83.1)
Moderate risk	-	17 (30.4)	5 (8.5)
High risk	-	8 (14.3)	4 (6.8)
Severe risk	-	3 (5.4)	1 (1.7)
Burnout prevalence			
Emotional exhaustion	69 (60)	35 (30.4)	34 (29.6)
Depersonalization	63 (54.8)	38 (33)	25 (21.8)
Low personal accomplishment	38 (33)	21 (18.2)	17 (14.8)
WEEI environment category			
Healthy	47 (40.9)	17 (14.8)	30 (26.1)
Risky	35 (30.4)	22 (19.1)	13 (11.3)
Toxic	33 (28.7)	17 (14.8)	16 (13.9)

Data presented as n (%), unless otherwise specified.

AUDIT-C = Alcohol Use Disorders Identification Test - Concise; PHQ = Patient Health Questionnaire; SD = standard deviation; WEEI = Work Environment Evaluation Instrument; WHO = World Health Organization.

* Among those who answered yes to professor abuse/harassment.

Several sociodemographic, personal, clinical, and work-related variables were associated with burnout symptom scores according to the MBI. The results of Mann-Whitney U tests comparing groups are shown in Table 2. Spearman’s rho correlations are shown in Table 3.

**Table 2:** Independent samples, Mann-Whitney U test

Variable	n	EE	DP	PA
Sex				
Female	59	30 (21)	8 (8)	37 (7)
Male	56	31 (19)	11 (6)	35.5 (8)
		p = 0.428	p = 0.002	p = 0.232
Physical activity WHO				
Yes	45	28 (22)	9 (8)	36 (7)
No	70	31 (18)	11 (7)	35 (7)
		p = 0.017	p = 0.034	p = 0.16
Workload				
< 40 hours/week	53	30 (21)	9 (7)	38 (7)
> 40 hours/week	62	30 (18)	11 (10)	35 (6)
		p = 0.160	p = 0.033	p = 0.072
Support from a preceptor				
Yes	99	30 (17)	10 (6)	36 (7)
No	16	37 (11)	12.5 (6)	32 (9)
		p = 0.025	p = 0.059	p = 0.002
Preceptor abuse/harassment				
Yes	66	32.5 (16)	11 (10)	37 (8)
No	49	22 (18)	8 (8)	35.5 (6)
		p < 0.001	p = 0.001	p = 0.516

Data presented as median (interquartile range).

EE = emotional exhaustion; DP = depersonalization; PA = personal accomplishment; WHO = World Health Organization.

**Table 3:** Correlations, Spearman’s rho

	EE	DP	PA
Age	-0.04	-0.22*	-0.06
Hours worked per week	0.19*	0.25^†^	-0.17
Years of medical experience	0.02	-0.25^†^	0.06
Relationship with family	-0.39^‡^	-0.36^‡^	0.29^‡^
Relationship with friends	-0.34^‡^	-0.22*	0.31^‡^
Relationship with preceptors	-0.53^‡^	-0.50^‡^	0.31^‡^
Relationship with peers	-0.26^†^	-0.27^†^	0.19*
Relation to institution	-0.57^‡^	-0.45^‡^	0.27^†^
Total WEEI score	-0.56^‡^	-0.48^‡^	0.32^‡^

EE = emotional exhaustion; DP = depersonalization; PA = personal accomplishment; WEEI = Work Environment Evaluation Instrument.

* p < 0.05; ^†^ p < 0.01; ^‡^ p < 0.001.

In the linear regression models (Table 4) the factors significantly correlated with EE (p < 0.05), in order of importance, were the nature of relations to the institutions themselves (beta -0.29; p < 0.05), the nature of the relationships with preceptors/supervisors (beta -0.27; p < 0.05), and the quality of the relationship with family (beta -0.26; p < 0.01). Regarding DP, the factors with the strongest correlations were the nature of the relationships with preceptors/supervisors (beta -0.38; p < 0.01), the quality of the relationship with family (beta -0.28; p < 0.01), and age (beta -0.18; p < 0.05). No factors were significantly correlated with PA in this sample according to the linear regression model.

**Table 4:** Linear regression models

	EE		DP		PA	
	R^2^ = 0.457		R^2^ = 0.440		R^2^ = 0.122	

	Beta	B (CI)	Beta	B (CI)	Beta	B (CI)
Age	-	-	-0.17*	-0.30 (-0.56 to -0.04)	-	-
Sex, male	-	-	0.12	1.46 (-0.39 to 3.3)	-	-
Physical activity (WHO)	-0.13	-3.10 (-6.7 to 0.51)	-0.14	-1.80 (-3.7 to 0.10)	-	-
Hours worked/week	-0.015	-0.36 (-3.9 to 3.2)	0.07	0.83 (-1.0 to 2.7)	-	-
Relationship with family	-0.26^†^	-3.07 (-0.99 to -0.5.15)	-0.28^†^	-1.69 ( -0.59 to -2.8)	0.18	1.05 (-2.2 to 0.15)
Relationship with friends	-0.06	-0.75 (-1.4 to 2.9)	-0.09	-0.55 (-1.7 to 0.60)	0.06	0.37 (-1.6 to 0.9)
Harassment/abuse by preceptor	0.09	2.04 (-1.8 to 5.9)	-	-	-	-
Support from a preceptor	-0.07	-2.32 (-3.1 to 7.7)	-0.07	-0.83 (-1.67 to 4.0)	0.16	2.7 (-0.44 to 5.9)
Relation to institution	-0.29*	-1.06 (-1.95 to -0.17)	-0.09	-0.16 (-0.62 to 0.29)	0.02	0.05 (-0.46 to 0.56)
Relationship with peers	0.11	0.43 (-.27 to 1.1)	-0.05	-0.10 (-0.47 to 0.26)	0.09	0.17 (-0.23 to 0.58)
Relationship with preceptors	-0.27*	-0.59 (-1.1 to -0.07)	-0.38^†^	-0.42 (-0.69 to -0.15)	0.08	0.08 (-0.21 to 0.38)

CI = confidence interval; EE = emotional exhaustion; DP = depersonalization; PA = personal accomplishment.

* p < 0.05; ^†^ p < 0.01.

According to the Kruskal-Wallis one-way analysis of variance (ANOVA) (Table 5), there were differences in burnout dimensions across groups based on the nature of the work environment (i.e., healthy, risky, or toxic), year of residence, family income, quality of sleep, and whether or not participants were in mental health treatment. In relation to the work environment, a healthy environment was related to lower EE and DP scores, and higher PA scores than risky and toxic environments (p < 0.001). Concerning residence year, individuals in their first year had higher DP levels compared to those in their second year (p < 0.05). Residents who had a monthly family income of US$ 387-775 had higher DP levels than those with a monthly income exceeding US$ 3875 (p < 0.05). Those with regular sleep quality exhibited higher EE levels than those who had good sleep quality (p < 0.05). Residents in regular psychiatric treatment or psychotherapy had higher EE than those not in treatment (p = 0.001).

**Table 5:** Kruskal-Wallis one-way ANOVA

	n	EE	DP	PA
Year of residency				
1	36	30 (18)	**11 (12)**	35 (5)
2	36	29 (15)	**10 (9)**	36 (9)
3	43	31 (20)	10 (7)	36 (7)
			**H(2) = 2.86; p = 0.009**	
Family income				
> US$ 3875	39	23 (21)	**8 (8)**	36 (8)
US$ 1917-3875	32	31 (15)	9 (6)	38 (6)
US$ 775-1937	33	33 (20)	10 (7)	35 (6)
US$ 387-775	11		**12 (9)**	34 (5)
			**H(3) = 12.36; p = 0.006**	
Work environment category				
Healthy	47	**21 (17)**	**7 (7)**	**39 (7)**
Risky	35	**30 (11)**	**11 (6)**	**35 (6)**
Toxic	33	**40 (12)**	**14 (11)**	**35 (6)**
		**H(2) = 31.90; p < 0.001**	**H(2) = 24.49; p < 0.001**	**H(2) = 13.74; p = 0.001**
Sleep quality				
Bad	10	38 (28)	10 (10)	34 (7)
Regular	48	**34 (17)**	11 (8)	36 (8)
Good	39	**28 (15)**	9 (6)	37 (8)
Great	16	22 (19)	10 (12)	37 (5)
Excellent	2	15 (29)	12 (12)	39 (7)
		**H(4) = 15.96; p = 0.003**		
Mental health treatment				
Psychiatrist only	7	32 (11)	7 (2)	36 (7)
Psychiatrist and @@psychotherapy	33	**37 (13)**	10 (8)	36 (9)
Psychotherapy only	40	31 (17)	10 (9)	36 (8)
No treatment	35	**22 (16)**	10 (10)	36 (7)
		**H(3) = 17.51; p = 0.001**		

Bold type denotes statistical significance (p<0.05).

EE = emotional exhaustion; DP = depersonalization; PA = personal accomplishment.

Regarding the risk of burnout symptoms according to work environment categories, the odds ratio (OR) for being positive for EE was 7.61 (95%CI 2.45-23.60; p < 0.001) and the OR for being positive for DP in a toxic environment was 5.82 (95%CI 2.38-14.25; p < 0.001). The OR for being positive for EE in a healthy environment was 0.17 (95%CI 0.07-0.39; p = 0.001) and the OR for being positive for DP in a healthy environment was 0.05 (95%CI 0.01-0.25; p < 0.001).

## Discussion

Our results portray a sample of psychiatry residents with high levels of emotional exhaustion and depersonalization and a worrying prevalence of screening positive for anxiety, somatization, depression, and suicidal ideation. Their risk of alcohol abuse and dependence according to the AUDIT-C is also alarming, especially among the men. Previous studies that evaluated psychiatry residents also show worrying rates of emotional distress among participants, namely symptoms of anxiety, depression, suicidal ideation, and use of psychotropic medications.^12,19^ In counterpoint to this, one fact that caught our attention was that, despite the high rates of emotional distress in our sample, the level of satisfaction with the profession and the feeling of personal accomplishment were both high.

To our knowledge, this is the first study that evaluates the role of interpersonal and institutional aspects as risk factors for burnout in psychiatry residents, and several of the aspects it revealed are worth mentioning. Eighty-six percent of the subjects reported they had had at least one preceptor/supervisor from whom they felt support. Nevertheless, 57.4% claimed to have suffered abuse/harassment from at least one preceptor/supervisor and 42.6% of these declared it had had a negative impact on their academic life. According to the WEEI, the environment was evaluated as healthy in 40.9% of cases, in 30.4% as risky, and in 28.7% as toxic. Moreover, according to the Kruskal-Wallis one-way ANOVA (Table 5), a healthy environment was related to lower EE and DP scores and higher PA scores than risky and toxic environments (p < 0.05). In the linear regression models, relations to the institutions themselves and the relationships with preceptors/supervisors were related to EE levels, and the relationship with superiors was also related to DP. The risk estimate tests equally support those results, showing a consistent impact of the nature of the relationships within the environment and of relations to the institutions themselves.

One can relate this finding to studies concerning psychiatry residents that have shown that burnout is related to reduced seeking of help from supervisors, reduced satisfaction with clinical faculty, lack of clinical supervision, and poorer perceived quality of supervision.^12,13,28-31^ These findings draw attention to potentially modifiable factors involving the influence of positive relations (with supervisors/preceptors and to the institutions themselves). By addressing the nature of the relations within the institutions, it should be possible to foster well-being in opposition to emotional distress during psychiatry residence. Hence, encouraging these values in training institutions may be one way to reduce burnout and associated distress in residents.

In our sample, a good quality of relationship with one’s family was correlated with lower levels of EE and DP. There are studies showing that residents with children have lower burnout scores compared to those without,^13,32^ but we did not find studies in the literature that specifically analyzed the association between burnout and the quality of the relationship with family. In any case, according to our data, the quality of one’s relationship with family outside the work environment appears to be an important factor, apparently protecting from the impact of toxic workplace experiences when the subject has a good relationship with family, or, on the contrary, augmenting the risk when they do not. Whether this factor remains significant in later phases of the career and other populations is yet to be elucidated.

In relation to DP, residents in the first year (31.3%) show more symptoms than those in the second year. We have found studies in the literature showing that being a resident in junior years of training is indeed related to greater levels of burnout.^29,30^ Particularly in psychiatry, where the first year is usually characterized by contact with inpatients, greater levels of depersonalization can be viewed as an attempt to protect oneself from the impact of the contact with powerful feelings triggered by patients. In this sense, having in mind the importance of the role of relationships with preceptors/supervisors in protecting residents from burnout symptoms, institutions should be even more active in facilitating the availability, quality, and humanity of supervision and support. Moreover, depersonalization may interfere with connecting to patients, which is a major concern in psychiatric treatment.

Also, according to our sample, male residents may be at even greater risk of suffering from depersonalization, although sex was not significant in the linear regression model. This finding suggests a possibly different pattern of burnout symptoms in males and females, and further studies, using larger samples and different populations should explore it. Studies about burnout and sex are controversial. Some have found that there is no association between sex and burnout, some that male residents had more symptoms, and others that female residents had higher levels of burnout.^12^ According to our data, since we analyzed the three dimensions of burnout separately, we believe that one hypothesis is that these controversies may in fact be caused by the existence of different patterns of burnout that are sex-based. Further studies should focus on evaluating burnout dimensions separately in both sexes to elucidate this matter. In our linear regression model, older age was correlated with fewer DP symptoms. This finding is in agreement with a large study conducted by Dyrbye et al.^33^ which found that mid-career physicians had much higher rates of burnout than older colleagues.

The residents practiced for a mean of 46.45 hours per week and 54.8% had another job besides the residency itself. Those who had another job worked a mean of 8 additional hours of work per week. Individuals with a workload exceeding 40 hours per week had higher levels of DP in our sample (p < 0.05). This specific variable was not included in the linear regression models, because we chose to use number of hours worked as a continuous variable. More studies are therefore needed to better evaluate the impact of this specific factor. However, this finding is in keeping with the literature showing that increased workload, long working hours, and insufficient rest are associated with burnout.^13,19,28,30^

The risk of alcohol abuse and dependence according to AUDIT-C was alarming in our sample, especially in men. About 50% of the male residents in our sample showed a moderate or worse risk of alcohol abuse. Our findings are consistent with the literature that shows a high prevalence of substance abuse among physicians and mainly in those presenting burnout. A study about the prevalence of substance use disorders in American physicians reported that of the 7,288 physicians who answered the questionnaire, 12.9% of male physicians and 21.4% of female physicians met diagnostic criteria for alcohol abuse or dependence, indicating that alcohol abuse or dependence is a significant problem among American physicians.^34^ Another study about burnout in Canadian psychiatry residents reported that the residents were more likely to engage in unhealthy coping mechanisms such as alcohol use, excessive shopping, or unhealthy eating.^29^

One intriguing finding is that, despite the high prevalence of emotional exhaustion in our sample (60%), the satisfaction with the profession is also very high (85.3%), as well as the feeling of being personally accomplished (67%). We are not aware of studies that have discussed this apparent contradiction. We believe that despite the difficulties associated with the work, helping other people can be very satisfying. In addition, entering a medical career usually takes a huge effort, and being in the profession one has chosen and identifies with can be related to a consistent sense of purpose that gives meaning to one’s life, regardless of the presence of EE and other psychiatric symptoms. Moreover, this might be related to specific characteristics of those individuals who make a career out of medicine and out of psychiatry in particular.^35^

There are several strengths of this study worth noting. Firstly, we were able to evaluate aspects not yet addressed in the literature concerning specific work environment features. Secondly, we were able to find potentially modifiable factors related to burnout. On the other hand, this study also has limitations. Firstly, the number of participants limits some analysis. It is possible that future studies with larger populations of psychiatry residents would find other associations. Secondly, this is a cross-sectional study, so we cannot infer causality between factors and outcomes. Thirdly, we did not find factors significantly associated with PA in the linear regression model. We believe this may be due to the reduced number of participants and/or the difficulty evaluating PA at the beginning of one’s career.

In conclusion, burnout in psychiatry residents is an important issue and was particularly related in our sample to work environment aspects (i.e., the nature of the relationships with preceptors/supervisors, and relations to the institutions themselves), as well as to the quality of the relationship with family. Also, there was a high prevalence of psychiatry symptoms and signs of emotional distress. It is important to emphasize that the institutional factors are modifiable, and institutions should develop strategies to enhance the healthy aspects of the environment, particularly the nature of the relations within their walls and the nature of the values fostered by the leaders. More studies are necessary to better understand these processes and to evaluate interventions developed to modify them.
